# Revolutionizing Renal Replacement: Current Advancements in Development and Transplantation of Bioengineered Kidneys

**DOI:** 10.3390/ijms27135879

**Published:** 2026-06-30

**Authors:** Rune Brulez, Marijn M. Speeckaert

**Affiliations:** 1 Department of Nephrology, Ghent University Hospital, 9000 Ghent, Belgium; rune.brulez@ugent.be; 2 Research Foundation-Flanders (FWO), 1000 Brussels, Belgium

**Keywords:** bioengineering, cell-on-scaffold, decellularization, recellularization, kidney, transplantation

## Abstract

The rising prevalence of chronic kidney disease represents a major global health burden. Limitations of current renal replacement therapies, including donor organ shortages, rejection, and dialysis-related complications, underscore the need for innovative treatment options. This narrative review assesses the feasibility of bioengineered kidneys as an alternative to current treatments by discussing advances in decellularization, recellularization, and the transplantation of cell-on-scaffold kidneys. We propose that the development of functional bioengineered kidneys follows a hierarchical, staged process, in which vascular patency is the primary prerequisite for graft survival, followed by partial restoration of glomerular filtration, with complete tubular function remaining the final and most challenging milestone. Perfusion-based whole-organ decellularization has made significant progress in preserving the extracellular matrix, enabling the production of acellular human kidney scaffolds. However, complete recellularization of whole kidneys has not yet been achieved. Nevertheless, partially repopulated kidney scaffolds have been shown to withstand physiological blood pressure, produce urine, and exhibit filtration in large-animal models. Complete endothelial coverage of the vascular network proved essential for preventing thrombosis after transplantation. Current work on bioengineered kidneys shows promising results regarding feasibility for clinical application. It is important to note that most of the included studies are proof-of-concept, characterized by small sample sizes and short observation periods. Although these findings are crucial for further research, they cannot be generalized, and larger trials are recommended. In addition to cell-on-scaffold kidneys, 3D bioprinting is a promising technique that could eliminate the need for donor scaffolds.

## 1. Introduction

Chronic kidney disease (CKD) is a progressive condition marked by persistent abnormalities in kidney structure or function lasting at least three months, with significant implications for patient health [1]. These abnormalities are often attributed to underlying conditions such as diabetes mellitus, arterial hypertension, obesity, and primary renal disorders [2,3]. Other causes of CKD include urinary tract obstruction, renal artery stenosis, toxins, medications, substance abuse, and congenital anomalies [4]. CKD affects approximately 13.4% of the global population and is a major contributor to morbidity and mortality worldwide, with rapidly rising prevalence. Epidemiological research indicates that CKD is expected to become the fifth leading cause of death by the year 2040 [3,5]. It is associated with a broad spectrum of complications, with cardiovascular disease the most prominent and clinically significant. More than half of deaths in patients with kidney failure are attributed to cardiovascular causes. Because early stages of CKD are often asymptomatic, it is frequently diagnosed at an advanced stage, limiting the effectiveness of early interventions and contributing to poor outcomes [3,6]. Current treatment strategies for CKD primarily aim to slow disease progression and reduce associated cardiovascular complications by optimizing blood pressure, lipid, and glycemic control in patients with diabetes, as well as general lifestyle adjustments [1,2,7,8]. Despite these measures, CKD remains largely irreversible, and a substantial proportion of patients ultimately progress to kidney failure, requiring renal replacement therapy (RRT) for survival. Kidney transplantation is considered the gold standard in RRT, offering superior survival rates and quality of life compared with dialysis [4,7]. Donor organs can be obtained from deceased and living donors.

Nevertheless, the availability of suitable donors remains insufficient to meet the growing demand. As a result, many patients are placed on waiting lists for extended periods and require dialysis as a bridging or long-term therapy. Another important drawback of kidney transplantation is the lifelong need for immunosuppressive therapy to prevent organ rejection. This therapy, while essential for preventing rejection, increases the risk of infections, malignancies, and metabolic disorders [9,10].

Several innovative approaches have been explored to overcome these limitations. Xenotransplantation has been proposed as a potential solution to the shortage of donor kidneys, involving the transplantation of organs from genetically modified animals into humans. Early studies of heart xenotransplantation have yielded limited success. Genetically modified pig kidneys have recently been transplanted into both brain-dead human recipients and a small number of living recipients, although long-term outcomes remain uncertain. Long-term outcomes remain uncertain due to immunological barriers, the risk of rejection, and ethical concerns. Another strategy aims not to replace the donor organ, but to modify the recipient immune system to achieve donor-specific tolerance. Protocols based on chimerism that combine kidney transplantation with donor hematopoietic stem cell or bone marrow transplantation have allowed the withdrawal of ongoing immunosuppression in a few kidney transplant recipients from living donors, including both HLA-identical and HLA-mismatched cases [11,12,13]. Some recipients, based on long-term follow-up studies, have been able to remain off anti-rejection drugs with stable graft function for years [11,12]. On the downside, these protocols are still constrained by the toxicity of conditioning, the complexity of the procedure, variable results, and limited applicability even in highly specialized centers [13]. Thus, immune tolerance induction is another important strategy for reducing or potentially eliminating lifelong immunosuppression. However, it does not address the shortage of transplantable kidneys and is completely different from organ bioengineering. Wearable dialysis devices represent another emerging strategy. Nevertheless, this technology primarily improves patient autonomy and well-being rather than increasing survival [14,15].

In recent years, regenerative medicine and tissue engineering have emerged as promising fields for developing alternative therapies. These approaches aim to repair or replace damaged tissues using human stem cells, which can be pluripotent, multipotent, or oligopotent, depending on their differentiation potential. This variety of options offers opportunities to use patient-derived stem cells in tissue engineering. In addition to tissue repair, these cells have shown strong potential to contribute to the formation of whole organs. In the context of kidney disease, several strategies have been investigated, including kidney organoids, cell-on-scaffold approaches, and three-dimensional (3D) bioprinting [15,16,17]. Kidney organoids rely on the self-organization of renal progenitor cells to form nephron and tubular structures. Although relatively easy to construct, they are not yet suitable for whole-organ development. Nevertheless, they provide valuable insights into kidney development and stem cell behavior that can inform other techniques that show greater promise for future applications. This is particularly true for the cell-on-scaffold approach, which has already been extensively investigated in other fields [17]. Over the years, tissue engineering has been explored for several organs. Decellularization and repopulation of scaffolds have shown promising results from the start, while 3D bioprinting is a more recent possibility, enabled by technological advances. The greatest challenge in developing a bioengineered kidney lies in its complex structure. Other organs, such as the trachea, bladder, or blood vessels, which are hollow structures, are relatively easy to repopulate with cells. This is not the case for the kidney, which contains more than 26 different cell types and has a complex architecture and function [18,19]. Both techniques rely on a scaffold that provides a 3D structure for organizing new cells. This scaffold can be obtained from a decellularized donor kidney or fabricated via 3D bioprinting using bioink [19]. The scaffold’s core function is to provide an extracellular matrix (ECM) that interacts with newly seeded cells, guiding their differentiation, organization, and function [20].

There are multiple approaches to whole-organ decellularization. The preferred approach for kidney decellularization involves perfusion with detergents and other chemical agents through the renal artery, vein, and ureter [21]. This method is well established and effectively removes cellular components while preserving structural integrity, yielding acellular scaffolds. These scaffolds are then repopulated with stem or progenitor cells via a similar perfusion-based approach. This recellularization process is currently far less effective than decellularization. The most significant drawbacks of incomplete recellularization are impaired function and post-transplant complications, such as thrombosis [22].

3D bioprinting is a rapidly evolving technology that fabricates synthetic scaffolds from bioinks composed of biomaterials [23]. Although this approach enables the fabrication of complex structures, a full-sized kidney scaffold has yet to be produced. Maintaining structural integrity and physiological function after printing also remains a major challenge. As a result, bioprinting currently serves as a tool for disease modeling and drug screening rather than for in vivo transplantation [15].

Clinical implementation of bioengineered kidneys would be a breakthrough in the treatment of kidney failure. It would not only benefit patients who need transplantation to avoid dialysis but also ideally reduce the substantial economic burden associated with conventional RRT [19]. Moreover, generating bioengineered kidneys using cells taken from patients themselves might lessen the immunological challenges of standard transplantation and, in principle, reduce lifelong reliance on immunosuppressive drugs. Still, total immune acceptance is not yet realized since bioengineered structures might contain immune-stimulating components of the ECM, leftover antigens from the scaffold, xeno-derived reagents used in manufacturing, or cellular populations that are not fully differentiated. In addition, tissue injury during implantation and remodeling may generate inflammatory signals that promote immune activation despite the use of autologous cells [24,25,26,27]. Therefore, the extent to which bioengineered kidneys can reduce or eliminate immunosuppressive requirements remains an important unanswered question that requires rigorous long-term investigation.

Despite advances in RRT, donor kidney shortages and graft rejection remain major challenges. This review examines whether bioengineered kidneys could offer a feasible alternative to address these challenges. The primary focus is on the technical aspects of cell-on-scaffold approaches and their preclinical outcomes. In addition, emerging technologies such as 3D bioprinting will be briefly discussed, along with key challenges, ethical considerations, socioeconomic aspects, and future perspectives.

## 2. Materials and Methods

This narrative review summarizes recent advances in the development and implantation of bioengineered kidneys. We searched PubMed, Web of Science, and Scopus for relevant literature using combinations of the keywords “tissue engineering”, “bioengineering”, “kidney”, “transplantation”, “decellularization”, and “recellularization”. Additional relevant publications were identified through manual screening of reference lists. Study selection was guided by relevance to the review’s objectives, with particular emphasis on decellularization, recellularization, vascularization, and the transplantation of bioengineered kidneys. Priority was given to experimental and preclinical studies that provided important insights into the development, functional assessment, and transplantation of cell-on-scaffold kidney constructs.

## 3. Results

### 3.1. Conceptual Framework: A Staged Model of Bioengineered Kidney Function

To facilitate interpretation of the rapidly expanding literature on kidney bioengineering, we propose a hierarchical three-stage model that captures the progressive acquisition of function in bioengineered kidneys. Although advances in decellularization, recellularization, and transplantation are often discussed as separate technical achievements, the ultimate objective is the restoration of an integrated organ capable of sustaining physiological renal function. Current evidence suggests that this process does not occur simultaneously across all renal compartments but instead follows a sequential, interdependent pattern in which specific functional milestones must be achieved before more advanced levels of organ performance can emerge [28,29,30]. Accordingly, the development of a bioengineered kidney can be viewed as the progression through three distinct yet interconnected stages: vascular patency, glomerular filtration, and tubular maturation.

The first step, vascular patency, is the most basic requirement for graft viability. Regardless of the quality of the decellularized kidney’s ECM architecture or its receptivity to recellularization, a kidney will remain non-viable in the absence of a functionally viable and perfusable vascular network. Immediately upon implantation of a decellularized kidney, circulating blood comes into contact with the non-endothelialized ECM, triggering a rapid coagulation cascade, platelet adherence to exposed collagen and tissue factor-rich surfaces, and subsequent thrombosis and graft rejection. This has been consistently observed in acellular transplantation experiments across different animal models, making it clear that restoring vascular integrity is the step that limits short-term graft retention [28,29]. Therefore, re-endothelialization of the vasculature must be seen as an essential step in kidney (bio)engineering; not only does the formation of a confluent, non-thrombogenic cell coat along the endothelialized wall (through the production of thrombo-modulating factors like nitric oxide and prostaglandin I2) reduce the likelihood of thrombosis, but endothelial cells also regulate vascular wall permeability, immune cell infiltration, and tissue perfusion. Re-endothelialization should, because of this, be seen less as a technical task and more as the biological enabler for all subsequent aspects of kidney growth and maturation. Progress has been made in this area, with improved long-term patency demonstrated by reintroducing cells using stem cell-derived endothelial subsets and an optimal scaffold structure [30,31,32].

Once stable vascular perfusion is established, bioengineered kidneys may progress to the second stage, glomerular filtration. The defining feature of this phase is the development of a functional filtration barrier that can produce ultrafiltrate but does not exclude circulating proteins and cellular components. In physiological terms, the glomerular filtration barrier comprises three distinct layers: the tips of fenestrated glomerular endothelial cells, the glomerular basement membrane, and podocyte foot processes, which are bridged by slit diaphragms. Reproducing this complex architecture poses a formidable challenge in tissue engineering. Nevertheless, several transplantation studies have demonstrated partial filtration function, including urine production, selective protein retention, and reduced hematuria, following transplantation of recellularized kidney scaffolds [28,31]. However, these findings primarily originate from proof-of-concept studies with small sample sizes and short observation periods and should therefore be interpreted as evidence of feasibility rather than of durable functional restoration. Importantly, these observations indicate that restoration of filtration capacity may be achievable before complete nephron reconstruction. This distinction is critical because it suggests that functional recovery follows a hierarchical rather than an all-or-none pattern. Partial glomerular function may emerge despite incomplete recellularization of other nephron segments. The recent demonstration of selective filtration in large-animal transplantation models further supports the concept that glomerular reconstruction is an achievable intermediate milestone on the path toward full organ regeneration [31]. However, current evidence also indicates that filtration remains quantitatively inferior to that of native kidneys and that sustained function over prolonged periods has yet to be demonstrated [28,31].

The third and most advanced stage is tubular maturation and nephron integration, which represents the ultimate challenge in kidney bioengineering. Although glomerular filtration appears to be predominantly reliant on the regeneration of structural specializations, the tubular system must rely on the diverse functions of epithelial cells derived from highly differentiated segments of the nephron, arranged in precise spatial patterns. The specialized transport capacities of the proximal tubule, the loop of Henle, the distal tubule, and the collecting duct each involve hundreds of membrane transporters, ion channels, metabolic pathways, and regulators. In addition, precise cellular positioning along the apical–basal polarity axis, formation of tight junctions, epithelial–mesenchymal interactions, and relationships with surrounding peritubular capillaries are imperative for a functional tubular system [33,34]. Re-establishment of tubular reabsorption and secretion likely presents a much greater biological challenge than restoration of filtration alone. In all current studies, tubular reabsorption and secretion remain incomplete, as directly reflected by the persistence of albuminuria and glucosuria, impaired tubular solute handling, and reduced urine-concentrating ability [28,31]. Epithelial cell differentiation, functional maturation, and spatial organization are promising, as indicated by recellularization strategies, but remain incomplete. The tubular component of a bioengineered kidney remains incapable of reproducing the complex homeostatic functions of the native kidney.

Importantly, these three stages should not be viewed as independent endpoints but as components of a hierarchical continuum. Progression through the model is sequential, with successful completion of earlier stages creating the biological conditions necessary for advancement to later stages. Vascular patency enables sustained tissue perfusion and cell survival, thereby supporting the development of glomerular structures. Functional glomerular filtration then supplies the ultrafiltrate required for tubular transport, and tubular maturation finally converts these basic filtration processes into integrated renal function, with the capacity to regulate fluid, electrolyte, acid–base, and metabolic homeostasis. This hierarchical structure provides a framework for understanding the field’s current state and explains why many transplantation experiments appear promising yet fail to produce a fully functional organ.

Beyond its descriptive utility, this staged model offers inspiration for practically significant next steps in research. It highlights that measuring success in functional renal restoration should not be limited to the final stage of renal function recovery; instead, it should also account for progress through biological milestones in a sequential manner. A system like this can make it easier to compare across studies, help set the most suitable experimental endpoints, and highlight the main obstacles to the clinical application of the results. On top of that, this perspective suggests that the next breakthroughs in this field will depend less on merely increasing the number of cell types involved and more on precise spatial arrangement, cell integration, vessel stability, and functional development within the engineered organ. From this viewpoint, the current progress in endothelialization, glomerular reconstruction, stem cell differentiation, organoid biology, and biofabrication can be seen as small but necessary stages that collectively lead to the long-term goal: bioengineered kidneys that may ultimately achieve sufficient structural and functional maturity to support clinically meaningful renal replacement [30,31,32,33,34].

Some evidence points to the feasibility of biological and technical kidney bioengineering, but it is far from establishing clinical feasibility. Most experiments so far have been concept demonstrations using very small animal populations or short-term large-animal transplantation models, and the outcome measures have been principally related to vascular patency, urine production, and partial filtration function. These accomplishments are markers on the road, but major issues must be solved for clinical translation to become a reality. For example, researchers have not yet achieved the recellularization of the whole nephron to the satisfaction of the field, including segment-specific epithelial differentiation and proper spatial organization. On top of that, it remains unclear how well tubular reabsorption and secretion work, how long the graft survives, how stable metabolic homeostasis is, and how kidney endocrine functions are recovered. Other concerns include immune compatibility, thrombosis prevention, long-term safety, scalable GMP-compliant manufacturing, and regulatory approval pathways. Thus, the current evidence should be regarded as showing preclinical biological feasibility rather than clinical readiness. Future research will need to demonstrate sustained organ function, long-term safety, and reproducible manufacturing before bioengineered kidneys are considered a therapeutic option for patients with kidney failure [26,27,28,30,31,35,36,37,38,39,40,41,42].

One major point to keep in mind when analyzing the current literature is that almost all transplantation studies to date are simply demonstration projects. Most of these studies involve only a handful of animals and monitor the transplanted organ for a relatively short period, often a few hours to a few days. Because of this, changes such as increased urine production, vasodilation, or signs of filtration should not be regarded as proof of a fully functioning, clinically relevant kidney transplant. On the contrary, these results highlight biological possibilities and offer significant molecular clues to guide further development. To verify a graft’s continued survival, long-term filtering ability, tubular function, and overall maintenance of the body’s internal environment, much larger experiments and longer observation periods will be necessary. Progress in two key domains is advancing the field toward the clinical implementation of bioengineered kidneys. These domains contribute sequentially to a hierarchical model of organ function, rather than independently, with vascularization preceding functional filtration and tubular maturation.

### 3.2. Human Kidney Decellularization

#### 3.2.1. Structural and Biochemical Preservation of the ECM

The primary objective of decellularization is to obtain an optimal ECM scaffold in which all cellular components and DNA fragments are removed while preserving the ECM’s architecture and biochemical characteristics. To achieve optimal decellularization, the most promising techniques from animal models are translated to human kidneys. The effectiveness of decellularization can be assessed using several techniques, such as histopathological staining, immunofluorescence, immunohistochemistry, scanning electron microscopy (SEM), and CT angiography (CTA). These methods assess the quality and quantity of ECM components required for cell attachment and signaling. Crucial components include collagen I and IV, fibronectin, elastin, laminin, and glycosaminoglycans. Optimal decellularization is crucial to minimize immune response after in vivo transplantation, while preservation of ECM components is important for cell growth and differentiation after recellularization [43,44]. One of the first studies demonstrating the feasibility of whole-organ decellularization of human kidneys was conducted by Orlando et al. Because more than 2600 kidneys are discarded for transplantation annually, the authors considered them an ideal platform for tissue engineering research. In this study, 10 human kidneys discarded for transplantation were decellularized by perfusion with 0.5% sodium dodecyl sulfate (SDS) for 48 h via the renal artery and ureter, followed by 5 days of rinsing with phosphate-buffered saline (PBS). Finally, scaffolds were evaluated using the previously mentioned assays. Key findings included preservation of ECM architecture, proteins, and the glomerular basement membrane. Moreover, 95% of the original DNA content was removed, and immunogenicity was reduced due to the complete removal of HLA class I and II antigens. The vascular network remained patent and compliant after perfusion under physiological pressure [43].

#### 3.2.2. Protocols and Reagents

Many protocols for perfusion-based chemical decellularization have been examined, using different detergents and varying perfusion durations. The most commonly used agents are ionic detergents, such as SDS, and non-ionic detergents, such as Triton X-100, which are often applied sequentially. SDS disrupts protein–protein interactions. Although effective at removing cells, it can compromise ECM structure. Triton X-100, on the other hand, causes cell membrane lysis by disrupting lipid–lipid and lipid–protein interactions. Both detergents are effective for decellularizing kidney grafts. Nonetheless, there are conflicting results regarding the optimal protocol. Shahraki et al. compared the two methods to determine which showed the greatest potential for further research. Overall, Triton X-100 showed better results than SDS. Although both detergents effectively removed cellular material from the scaffold, Triton X-100 achieved a higher degree of cell nucleus elimination. Furthermore, Triton X-100 fully preserved the ECM architecture, the integrity of the vascular network, and the glomerular basement membrane. In contrast, SDS treatment resulted in minimal disruption of the basement membrane and loss of glomerular ECM organization [45].

Nevertheless, SDS remains widely used as a detergent. Khosropanah et al. aimed to optimize the SDS exposure protocol, focusing on perfusion timing and SDS concentration. The ultimate goal was to decellularize a human kidney as quickly and efficiently as possible while preserving optimal ECM. Six experimental groups differing in SDS concentration (0.1%, 0.3%, and 0.5%) and perfusion duration (24 and 48 h) were compared. Perfusion decellularization was performed via the renal artery using a peristaltic pump at controlled flow rates. All protocols were terminated by flushing the scaffolds with Triton X-100 to remove residual ionic components. Overall, the results demonstrated that exposure to 0.1% SDS for 24 h achieved complete decellularization while preserving the ECM’s biochemical composition. Other protocols were more destructive. In addition, higher SDS concentrations, such as 1%, eliminated ECM growth factors and signaling molecules essential for repopulating the scaffold [44].

Given the suboptimal quality of SDS decellularization, recent studies have also focused on a promising new detergent, sodium lauryl ether sulfate (SLES). This method has yet to be examined in human kidneys but has shown promising results in rat kidneys and hearts. Kawasaki et al. compared decellularization with 1% SLES and 1% SDS. Although both detergents preserved collagen I and IV, laminin, and fibronectin similarly, 1% SLES performed better at preserving ECM and glycosaminoglycan content. This led to less inflammation and thrombogenicity after mesenteric implantation in rats. These results indicate that SLES is a less aggressive detergent than SDS [46].

Keshvari et al. conducted a similar experiment. A total of six rat kidneys were decellularized: three with 1% SDS and three with 1% SLES. Deep characterization of the scaffolds showed that ECM in the SLES group was significantly better preserved than in the SDS group. Subsequently, scaffolds from both groups were seeded with human umbilical cord mesenchymal stromal/stem cells (hUC-MSCs) and allotransplanted into the rat’s back muscle. This transplantation was performed solely to observe potential scaffold rejection or scaffold-related toxic effects. One month after transplantation, scaffolds were explanted and evaluated. There were no signs of rejection, and only sparse leukocyte infiltration was observed in the grafts. H and E staining showed new vascularization in both SDS and SLES scaffolds [47].

The key mechanistic differences between ionic and non-ionic detergents help explain the inconsistent results across studies. For example, SDS, an ionic detergent, disrupts protein–protein interactions, disrupts cellular membranes, and denatures ECM proteins, particularly glycoproteins and growth factor-binding domains. This may explain why scaffolds treated with SDS exhibited a loss of glomerular ECM organization and signaling capacity. By contrast, Triton X-100, a non-ionic detergent, primarily disrupts lipid interactions, thereby preserving protein structure. This is why the integrity of the basement membrane and vascular architecture is best preserved with Triton X-100, as evidenced by multiple published studies. However, Triton X-100 alone usually cannot completely remove cellular debris and DNA, which might lead to higher immunogenicity after transplantation. As a result, many protocols have, over time, combined SDS and Triton X-100 to achieve a good balance between efficient decellularization and ECM preservation. The lack of agreement among the studies might be due to differences in how they weigh ECM preservation against complete decellularization, and these differences may extend beyond technical factors. This variability highlights the lack of standardized experimental protocols and emphasizes the need for harmonized outcome measures. Without consistent endpoints, direct comparison between studies remains difficult, which limits the ability to identify optimal strategies for clinical translation. It also poses a major dilemma for the field: should ideal scaffolds prioritize preserving the ECM as much as possible (which calls for relatively gentle detergents such as Triton X-100 or SLES), or should the primary goal be the complete removal of cellular components (which is achieved using SDS-based methods)? Subsequent research should conduct head-to-head comparisons of these methods using common functional criteria such as thrombogenicity, recellularization capability, and long-term graft survival.

### 3.3. Human Kidney Recellularization

In contrast to well-established decellularization protocols, recellularization methods are still under investigation to determine the most effective approach. As in decellularization, cell seeding is performed by perfusion through the renal artery, vein, or ureter. Various cell types, including human induced pluripotent stem cells (hiPSCs), human embryonic stem cells (hESCs), human umbilical vein endothelial cells (HUVECs), and renal stem/progenitor-like cells, can be used for this purpose. After seeding, stem cells differentiate into multiple renal lineages due to the essential components of the ECM. Research on human kidney recellularization remains scarce. Nevertheless, studies conducted in non-human primates already provide valuable insights into this topic.

Batchelder et al. [48], for example, compared the differentiation of hESCs seeded within whole or sectioned rhesus monkey kidney ECM scaffolds to that of stem cells cultured in suspension as embryoid bodies. Moreover, stem cells were seeded into the scaffold, with or without additional growth factors or cytokines, to assess differences in differentiation. This was evaluated by measuring the expression of genes used as mesodermal and renal lineage markers with qPCR. Results showed that cells preferentially migrated to renal pyramids and medullary rays, with fewer cells observed in glomeruli or outer cortical tubules. Cells were distributed in a relatively uniform manner, but full repopulation of the endothelium or parenchymal compartments was not achieved. Neither whole kidney scaffolds nor kidney ECM sections were fully repopulated. This suggests that perfusion and cell infiltration are unlikely to be limiting factors, provided the need for additional signaling factors is met.

Regarding the degree of differentiation, all mesodermal and renal development markers were upregulated in whole kidneys and kidney sections compared with embryoid body cultures. For example, the mesodermal gene *BRY* was upregulated by more than 80-fold in both conditions. This confirms the presumed importance of ECM components in cell differentiation during kidney development. Additionally, stem cell differentiation was compared between a natural ECM scaffold and a new, biologically inert polysaccharide scaffold. Two protocols, each using different growth factors, were applied to both scaffolds to assess the synthetic variant’s potential. Both scaffolds yielded similar results in 3D tubule development and in the expression of markers associated with renal progenitor, proximal tubule, endothelial, and collecting duct cell populations. These findings suggest that additional growth factors play a more critical role than ECM components in stem cell differentiation. This shows potential for future research on synthetic scaffolds to eliminate the need for donor organs.

One of the few studies on human kidney recellularization was conducted by Bombelli et al. The study aimed to assess whether nephrosphere cells, composed of renal stem and renal progenitor-like cells, could repopulate nephron segments in human decellularized kidney slices. Nephrosphere cells were cultured on these slices in basal, endothelial, or epithelial medium to assess the effects of the medium on cell differentiation. Cells cultured in basal medium differentiated into proximal and distal tubular cells and endothelial cells. Endothelial and epithelial media promoted differentiation toward endothelial and epithelial lineages, respectively. Cell differentiation was confirmed by histology and expression of tissue-specific markers. These findings suggest that nephrosphere cells are a viable option for scaffold repopulation and warrant further exploration for whole-kidney recellularization [49].

### 3.4. In Vivo Transplantation in Animal Models

Studies on the technical aspects and in vitro outcomes of cell-on-scaffold kidneys are essential for developing a standard protocol that yields optimal outcomes for subsequent preclinical and clinical trials. Several preclinical trials have already been conducted in both small- and large-animal models. Protocols and key findings from these preclinical transplantation studies are summarized in Table 1.

Early proof of principle for functional orthotopic transplantation of bioengineered kidneys was demonstrated by Song et al. [28]. Cadaveric rat kidneys were decellularized by perfusion with 1% SDS and Triton X-100 through the renal artery, then repopulated with HUVECs and rat neonatal kidney cells (NKCs) via the renal artery and ureter, respectively. After orthotopic implantation in rats, the kidneys were perfused by host circulation and produced rudimentary urine. Nevertheless, their function was short-lived and incomplete. Urine production, creatinine clearance, and urea excretion were observed but were significantly lower than in native kidneys. Albuminuria and glucosuria were also observed, indicating insufficient filtration and reabsorption. Nevertheless, this study remains an important proof-of-concept for the orthotopic implantation of functional cell-on-scaffold kidneys.

Meanwhile, other studies focused on scaling up from rodent models to larger animals, such as pigs and sheep, to generate findings with greater clinical relevance. Early trials used acellular scaffolds, without prior cell seeding, to assess surgical feasibility, biocompatibility, and vascular integrity [29,50,51]. These studies reported similar results across animal models. Peloso et al. [50] removed and decellularized 26 kidneys from male Lewis rats. A total of 19 of these were thoroughly characterized using cellular seeding tests, DNA quantification, histological and immunofluorescent analyses, and scanning electron microscopy to evaluate preservation of ECM components and the quality of decellularization. The seven remaining kidneys were transplanted into rats that had undergone unilateral nephrectomy. The rats were sacrificed 7 days post-transplantation, and the scaffolds were histologically analyzed. All scaffolds remained morphologically intact. However, all vascular structures were occluded by thrombi.

Orlando et al. [29] conducted a similar experiment in porcine models. Pig kidneys were harvested and decellularized, then characterized in vitro using histologic analysis, pressure testing, and vascular assessment with fluoroscopy. Four decellularized kidney scaffolds were transplanted into pigs, who were sacrificed 2 weeks post-transplant. No leaks or other adverse effects were observed during this period. As expected, all renal arteries and veins were occluded by thrombi. Nevertheless, the gross anatomy of the scaffolds was well preserved.

A more recent study by Kajbafzadeh et al. [51] used sheep to compare the effects of two decellularization protocols on vascular network preservation. Sheep kidneys were decellularized with 1% Triton X-100 and 0.5% SDS (protocol 1) or 1% SDS (protocol 2). They were characterized with SEM and CTA to evaluate the vascular tree. CTA images showed vascular leakage within the scaffolds from protocol 2 but not from protocol 1. After in vitro characterization, four kidneys (two from each protocol) were transplanted into four sheep to observe in vivo outcomes. Sheep who received a scaffold from protocol 1 survived for 10 and 12 h, respectively, and died due to respiratory failure or intestinal perforation. Sheep who received a scaffold from protocol 2, on the other hand, died 2 h post-transplantation due to hypotensive shock. This was most likely due to vascular leakage and internal hemorrhage, consistent with prior CTA findings.

All these studies showed that acellular ECM scaffolds withstood systemic blood pressure and remained intact throughout the implant period. First of all, although thrombosis was observed in all models due to the lack of endothelial coverage, these trials demonstrated the potential for implanting acellular scaffolds in larger animal models and provided a platform for further research with repopulated scaffolds. Even though the transplantation studies were designed differently, a thorough comparison shows the same findings. In fact, for rodent, pig, and sheep models, there are three widely reproducible trends. Although acellular scaffolds maintain structural integrity at physiological blood pressure, they thrombose quickly after reperfusion in all cases, usually within minutes to days. There is a logical explanation for the rapid formation of clots in acellular scaffolds: the blood is directly exposed to the non-endothelialized extracellular matrix. When it comes to blood vessels, the endothelial cells constitute the inner lining and act as a medium producing nitric oxide, prostacyclin, thrombomodulin, and a heparan sulfate-rich glycocalyx, which collectively inhibit platelet activation and coagulation [53,54]. Decellularization disrupts this protective endothelial lining and its glycocalyx, thereby exposing collagen, laminin, fibronectin, and other matrix proteins to the blood [53]. Platelets quickly bind to the exposed collagen via glycoprotein VI and integrin 21, while the von Willebrand factor is responsible for platelet attachment through glycoprotein Ib-IX-V, mostly under conditions of high shear stress [53,55]. This platelet–collagen interaction induces platelet activation, leading to granule secretion, thromboxane A2 production, and recruitment of additional platelets, thereby enlarging the thrombus. At the same time, tissue factors originating from damaged vascular surfaces and the exposed matrix can initiate the extrinsic coagulation pathway, whereas contact of blood with non-endothelialized biological surfaces may activate the intrinsic pathway through factor XII [56,57]. The thrombin produced plays a central role in accelerating platelet activation and converting fibrinogen into fibrin, which acts as a stabilizing agent for the thrombus [57]. In addition to coagulation activation, complement pathways can also come into play during blood–material interactions, leading to the formation of an inflammatory microenvironment that further promotes platelet activation, leukocyte infiltration, endothelial dysfunction, and tissue factor expression, thereby establishing a two-way connection between innate immunity and thrombosis [56,58]. Disrupted intragraft hemodynamics further worsen these occurrences. High shear stress promotes platelet adhesion via the von Willebrand factor, whereas low-flow and stasis areas within microvascular networks that are not yet fully endothelialized favor fibrin accumulation and clot stabilization [53,59]. That means that thrombosis in bioengineered kidney scaffolds should not be interpreted solely as the consequence of insufficient endothelial coverage, but rather as the end product of a complex chain of events starting with the exposure of the extracellular matrix, platelet adhesion, complement activation, the coagulation cascade, loss of the glycocalyx, and finally, disturbed shear conditions. This multifactorial disease mechanism also explains why anticoagulation therapies used in preclinical transplantation experiments, such as systemic heparinization, aspirin, and clopidogrel, can extend the time the graft remains open but still cannot fully compensate for the lack of endothelialization [30,31]. Long-term vascular survival may depend on a combined approach that includes full endothelial coverage, re-establishment of glycocalyx-like surface properties, local anticoagulant scaffold modification, optimization of vascular flow dynamics, and careful, well-planned systemic antithrombotic treatment [30,31,56,58]. Thirdly, functional outcomes such as urine production and filtration were demonstrated. However, these effects remain limited and short-lived. Importantly, these observations were generated in a limited number of experimental animals and were generally assessed over hours to days rather than weeks or months. Consequently, urine production and filtration should be viewed as preliminary indicators of biological activity rather than as evidence of sustained renal replacement capacity. A more precise use of functional terms is necessary when explaining these results. Producing a urine-like fluid only indicates that fluid is emitted from the ureter or the collecting system after the graft is reperfused; by itself, it does not indicate physiological renal function. This fluid can be a sign of vascular leakage, passive ultrafiltration, incomplete tubular handling, or actual glomerular filtration [60,61]. Partial filtration means that plasma water and small solutes have partially moved across a newly formed filtration interface. However, it does not prove that filtration is quantitatively sufficient, size-selective, or continuous. Selective filtration implies a higher level of function and requires evidence that blood cells and large plasma proteins are retained while water and small solutes reach the urinary space, for instance, by a reduction in hematuria or proteinuria [62,63]. Still, even selective filtration does not demonstrate renal replacement function if tubular transport is absent or underdeveloped.

More than urine production is required for genuinely meaningful kidney function on a clinical level. A functioning kidney combines glomerular filtration with tubular reabsorption and secretion across different nephron segments. Proximal tubule cells must recover filtered glucose, amino acids, bicarbonate, phosphate, sodium, and water, while eliminating organic anions and cations [64]. The loop of Henle, the distal tubule, and the collecting duct must establish a cortico-medullary osmotic gradient, maintain sodium and potassium balance, respond appropriately to aldosterone and vasopressin, and concentrate or dilute urine according to the body’s needs [65,66]. Therefore, the persistence of albuminuria, glucosuria, dysfunctional solute handling, or an inability to concentrate urine still indicates incomplete tubular maturation, even if urine-like fluid production or partial filtration has occurred [28,31,64,66].

Beyond that, renal replacement depends on endocrine and metabolic functions, which are rarely evaluated in existing bioengineered kidney research. Natural kidneys regulate blood pressure through renin secretion, mineral metabolism by activating vitamin D, erythropoiesis by producing erythropoietin, acid–base balance by ammoniagenesis and bicarbonate reclamation, and detoxification by clearing and secreting endogenous metabolites and xenobiotics. Therefore, a transplantable bioengineered kidney should be benchmarked on sustained metabolic homeostasis rather than short-term urine production, including stable extracellular volume, electrolyte balance, acid–base status, nitrogenous waste clearance, endocrine activity, and long-term graft viability [67,68]. This means that different functional outcomes, such as urine-like fluid generation, partial filtration, selective filtration, tubular transport, endocrine function, and durable systemic homeostasis, should be reported by future researchers, with clear distinction among them. In contrast to glomerular filtration, which can be partially restored through structural reconstruction, tubular function requires highly specialized cellular differentiation and tight junction formation, representing a more advanced stage of organ integration. Importantly, the extent of functional restoration did not correlate strongly with the number of different cell types used for recellularization. Notably, the functional outcomes reported across studies do not scale proportionally with the number of cell types used for recellularization. Such observations underscore a fundamental principle in developmental biology and tissue engineering: mere cellular diversity cannot replicate an organ’s function. The kidney is much more than a mere sum of 26+ cell types; in fact, it is a highly structured organ in which cell determination, cell localization, interactions among cells and the matrix, blood vessel integration, and cell communication are harmoniously coordinated across multiple spatial levels. During embryonic nephrogenesis, the growing kidney (ureteric bud) and the kidney precursor tissue (metanephric mesenchyme) communicate to form the various cell types, tubes, blood vessels, and supporting cells of the kidney by delivering morphogen gradients wingless/integrated (WNT), fibroblast growth factor (FGF), bone morphogenetic protein (BMP), notch, and vascular endothelial growth factor (VEGF) signaling, as well as retinoic acid pathways [69,70,71,72]. Reproducing this developmental architecture in an engineered organ remains substantially more challenging than generating individual renal cell populations.

The functional performance of the native kidney depends on highly ordered microanatomical relationships. To form a functional filtration barrier, podocytes must be precisely aligned with glomerular endothelial cells, separated by a specialized glomerular basement membrane. Many features of proximal tubular epithelial cells, such as apical–basal polarity, a brush border, and the assembly of tight junctions, as well as their close association with peritubular capillaries, support their primary function of vectorial transport. The spatial organization of loops of Henle, vasa recta, and collecting ducts in the cortico-medullary area is essential for creating a countercurrent concentration gradient that enables urine concentration. Even minor disruptions in these spatial relationships can profoundly impair organ function despite the presence of otherwise differentiated cell populations [62,67,73,74].

Equally important is the concept of microenvironmental integration. Cells in native kidneys are constantly exposed to biochemical and biomechanical influences from the extracellular matrix, other cells, blood flow, oxygen, and soluble signaling molecules. The extracellular matrix serves not only as a physical scaffold but also as a signaling medium that regulates cell adhesion, migration, differentiation, proliferation, and survival through integrin-based mechanotransduction pathways [20,75]. A continuous exchange of information among endothelial cells, pericytes, fibroblasts, immune cells, and epithelial cells is essential for tissue homeostasis. So, besides simply replenishing each compartment, the recellularization process, if successful, also entails constructing these complex multicellular signaling networks.

Current recellularization strategies often achieve partial engraftment of individual cell populations yet fail to establish the spatial precision and multicellular integration required for physiological organ function. This limitation may explain why increasing the number of seeded cell types has not consistently led to proportional improvements in graft performance. Rather than a simple cell-delivery problem, whole-kidney recellularization should be viewed as a challenge to recreate developmental patterning, tissue self-organization, and multicellular ecosystem dynamics within a three-dimensional scaffold. Future progress will likely depend on technologies that control cellular positioning with high spatial resolution, including developmental bioengineering approaches, organoid-guided recellularization, spatially patterned biomaterials, advanced bioreactor systems, and high-resolution bioprinting platforms. Ultimately, successful kidney bioengineering will require reconstructing not only the kidney’s cellular composition but also its hierarchical structural organization and functional microenvironment.

This suggests that correct spatial organization and microenvironmental integration are more critical determinants of graft performance than cellular diversity alone. Following these trials, more recent studies have focused on vascular patency and kidney function after transplantation in large animal models. Uzarski et al. [30] and Lo et al. [31] conducted two important studies on these topics. Uzarski et al. [30] were the first to achieve vascular patency of re-endothelialized kidney scaffolds for up to 7 days after transplantation in vivo. A total of nine kidney grafts re-endothelialized with HUVECs were transplanted into pigs. All of them received methylprednisolone to suppress host immune rejection of the grafts, since the grafts were repopulated with human cells. Five of them received clopidogrel and aspirin daily from day 1 postoperatively; four did not. One of the pigs died due to excessive perioperative bleeding. Two others, who did not receive anticoagulation drugs, showed a lack of graft perfusion due to thrombosis. Thrombosis resulted from graft movement and vessel torsion, complications of the surgical techniques. Of the six grafts that showed no complications, five remained patent for the 7-day transplantation period. One of those did not receive anticoagulation drugs during the whole period of transplantation. Perfusion was measured through angiography. Differences in perfusion compared to native kidneys were not statistically significant [30]. Although these findings represent an important advance in vascular engineering, the study was designed primarily to demonstrate feasibility, included a limited number of transplanted grafts, and evaluated outcomes over only seven days. Therefore, conclusions regarding long-term vascular stability or clinical translatability remain premature. A second important finding of this study was the turnover of HUVECs and the invasion of porcine endothelial cells during the transplantation period. By day 7 post-transplantation, explanted grafts showed no residual HUVECs on immunofluorescence staining. This observation implies that the primary role of seeded endothelial cells may be transient, serving as an initial antithrombogenic interface rather than a permanent cellular component. The most recent and detailed large-animal transplantation data come from Lo et al. [31]. In this study, decellularized porcine scaffolds were repopulated with HUVECs on the vascular side and glomerular outgrowth cells (GOCs) on the urinary side. These GOCs were expanded from human donor tissue and chemically induced toward a podocyte phenotype. Cell seeding was performed in a closed perfusion bioreactor, with repeated HUVEC seedings through the renal vein and artery (150 million cells per seeding) and a single large GOC ureteral seeding (≈500 million cells) under negative vacuum pressure (−40 mm Hg) to reach cortical glomeruli. After a 24-day culture protocol, including additional HUVEC seedings and podocyte differentiation, the grafts were assessed in vitro using normothermic perfusion with porcine blood. Key quantitative endpoints in this part of the study were in vitro urine production comparable to that of native perfused kidneys, and significantly lower proteinuria and hematuria in bi-culture (HUVEC + GOC) grafts versus HUVEC-only controls. These outcomes indicate size-selective filtration mediated by podocyte function in bicellular grafts. For the following part of the study, a total of five bi-culture grafts were heterotopically implanted in pigs across three implant sessions (*n* = 2, *n* = 2, and *n* = 1, respectively). The implantation protocol was refined after each session, for example, by reducing systemic heparin dosing. Urine flow, hematocrit, and protein levels were measured at 30 and 60 min post-transplantation. Average urine flow during in vivo perfusion was 1.26 mL/min ± 1.3 across the three sessions. Urine hematocrit and protein levels decreased across sessions, reflecting improvements in the transplantation protocol. Implant session 3 demonstrated sustained filtration for up to 5 h post-transplantation, with a remarkably low urine hematocrit of approximately 1% and low urine protein relative to serum protein. This can be interpreted as evidence of blood filtration consistent with glomerular function [38]. These findings are consistent with partial restoration of glomerular filtration. In any case, we must be careful about how we interpret these results. The experiment included only five transplanted grafts, and the functional evaluation was conducted shortly after transplantation. The fact that the graft could produce urine and perform selective filtration is very promising. However, these effects are insufficient to demonstrate that kidney function was maintained or that the graft remained viable over time.

Few transplantation studies have used completely recellularized kidneys. This reflects the previously mentioned challenge of achieving optimal recellularization of whole kidneys. Geng et al. [52] sought to address this issue in two ways. First, structural ECM preservation was optimized by adding dextran to minimize collagen loss. In addition, the growth microenvironment was optimized by adding naphthalenephenylalanine-phenylalanine-glycine-arginine-glycine-aspartic (Nap-FFGRGD) to the acellular scaffold to promote adhesion and proliferation of the seeded cells. Recellularization was performed with iPSC-derived renal progenitor cells, which showed potential for self-assembly by repopulating specific nephron segments. Immunofluorescence detected podocyte and renal tubule markers in glomeruli and renal tubules, respectively. After transplantation, the repopulated scaffolds were immediately perfused by the host circulation, with no vascular leakage and an intact glomerular basement membrane.

### 3.5. Optimizing Vascularization

Because acellular scaffolds consistently induce thrombosis, efforts have focused on optimizing endothelial coverage of the vascular network. Early studies demonstrated successful endothelialization with HUVECs, but post-transplant cell adherence remained suboptimal [31,52]. To address this limitation, Leuning et al. [32] developed an improved re-endothelialization strategy using both rat and human kidneys. In this study, two human kidneys discarded for transplantation were decellularized with 1% SDS for 5 days, followed by 1% Triton X-100, to generate scaffolds. Recellularization was performed with hiPSC-ECs rather than HUVECs. Additionally, two key modifications were introduced. The vascular matrix was preloaded with vascular endothelial growth factor (VEGF) and angiopoietin-1, and a novel perfusion approach was implemented. Both the renal artery and vein were simultaneously perfused with hiPSC-ECs in an airtight bioreactor. This resulted in complete endothelial coverage, including peritubular and glomerular capillaries. Notably, these scaffolds withstood 25 min of in vitro perfusion with human whole blood, whereas acellular scaffolds occluded within 5 min. The perfusion duration was kept short to preserve the scaffolds for immunohistochemical analysis.

Complementary work by Wang et al. [76] focused on reducing thrombogenicity by immobilizing heparin onto the scaffold via a collagen-binding peptide (CBP). Heparin inhibits platelet adhesion and thrombus formation resulting from interactions between blood and the exposed ECM. Among unmodified scaffolds, free heparin scaffolds, and CBP-heparin-modified scaffolds, the latter exhibited minimal platelet adhesion, as observed by confocal and scanning electron microscopy. Furthermore, after recellularization with HUVECs and 7 days of perfusion, CBP-heparin scaffolds showed enhanced endothelial adhesion and coverage, particularly within glomerular regions.

Building on these findings, subsequent research combined VEGF with immobilized heparin to enhance neovascularization in vivo [77]. When implanted into rat renal capsules, these modified scaffolds showed significantly greater endothelialization, as assessed by CD31 staining, particularly on days 7 and 14 post-transplantation, compared with control groups (unmodified, VEGF, or heparin-modified scaffolds).

## 4. Discussion

Significant progress has been made toward bioengineering functional human kidneys through advances in decellularization, recellularization, and transplantation research. When interpreted through the proposed hierarchical model, these advances reveal a sequential progression of technical and biological challenges that must be overcome to achieve full organ functionality. Although the complete regeneration of fully functional cell-on-scaffold kidneys remains unrealized, recent studies have demonstrated crucial milestones that collectively indicate the growing feasibility of this approach. The development of effective decellularization protocols for whole human kidneys marks a major milestone in tissue engineering. Findings from animal models have been successfully translated to human organs, enabling the decellularization of complex structures such as the kidney. In particular, perfusion-based decellularization using both ionic and non-ionic detergents has emerged as the most effective approach [78]. Agents such as SDS and Triton X-100 consistently remove cellular components while preserving key ECM components, including glycosaminoglycans and structural proteins such as collagen, laminin, elastin, and fibronectin. From the perspective of the proposed model, priority should be given to strategies that facilitate progression from early vascularization (stage 1) to functional nephron integration (stages 2 and 3), particularly by promoting epithelial maturation and spatial organization.

The development of well-preserved ECM scaffolds has facilitated the exploration of recellularization strategies. Although studies on human kidney scaffold recellularization are scarce, animal models have provided valuable insights into this process. While complete recellularization remains unrealized, studies demonstrate that seeded cells can attach, survive, and differentiate toward renal lineages. Different stem cell types have been used for this purpose. Re-endothelialization was primarily performed using HUVECs or hiPSC-ECs, whereas repopulation of glomeruli and tubules was primarily performed using nephrospheres and renal progenitor-like cells derived from donor kidneys or hESCs.

Animal transplantation studies have demonstrated the feasibility of bioengineered kidneys in vivo. These proof-of-concept experiments provide an essential foundation for clinical translation. By demonstrating in vivo filtration and urine production across rodent and large-animal models, these studies yielded valuable insights for human translation. Early acellular scaffold transplantation demonstrated biocompatibility, reperfusion, and resilience to physiological blood pressure. Nevertheless, thrombosis across all these trials underscored the necessity of endothelial coverage of the vascular network. Scaffolds achieved this by repopulation with HUVECs, which maintained vascular patency for up to 7 days post-transplantation. One study reported HUVEC turnover.

The most critical milestone was achieved by Lo et al. [31], who demonstrated partial kidney function in a large-animal model. Grafts seeded with both HUVECs and glomerular outgrowth cells exhibited sustained perfusion and urine production with selective filtration, consistent with glomerular function after implantation in pigs. This study successfully translated the early findings from Song et al. [28] into a more clinically relevant porcine model, a crucial step toward the feasibility of bioengineered kidneys in humans.

Despite significant progress in bioengineering, several challenges remain in developing a functional cell-on-scaffold kidney. First, there are conflicting results regarding the optimal decellularization protocol. Several studies used 1% SDS as a standard detergent, whereas Khosropanah et al. [44] recently demonstrated that 0.1% SDS is significantly less destructive yet equally effective for decellularization. This makes it difficult to compare different studies. Moreover, the recellularization of scaffolds poses the biggest challenge [79]. Suboptimal cell seeding may result from insufficient perfusion. Alternatively, inadequate signaling and growth factors may limit cell attachment and differentiation. These findings suggest that current ECM signaling is insufficient for complete repopulation. Additional growth factors may be required to enhance this process [80]. Alongside recellularization, preventing thrombosis after transplantation has also been a major focus. Optimizing endothelial coverage has proven essential for long-term graft survival. Significant progress has been made by modulating scaffolds with immobilized heparin and VEGF. However, results have been assessed only by in vitro assays and heterotopic implantation. Results from orthotopic implantation using these new techniques are lacking.

A final challenge in tissue engineering is choosing the cell type to repopulate scaffolds. Multiple types of stem and progenitor cells, listed in Table 2, have been used in various trials. It remains unclear which type will be most suitable for optimal recellularization [81]. However, certain approaches are gaining popularity; for example, the use of HUVECs for re-endothelialization. When investigating the most suitable cell type, researchers should also consider cell availability. Cells should come from a sustainable and ethically justified source. hiPSCs, for example, theoretically provide an unlimited cell source. Nevertheless, certain drawbacks, discussed further below, currently limit their use. Cell types used in current trials are often derived from donor tissue, such as renal progenitor cells. These are present only in small amounts in kidney tissue, making them difficult to use in large clinical settings. Embryonic stem cells also show promising results, but they raise serious ethical concerns. Finding a balance among the advantages, disadvantages, availability, and ethical aspects of stem cell use will be a major challenge in the future.

**Figure 1:**
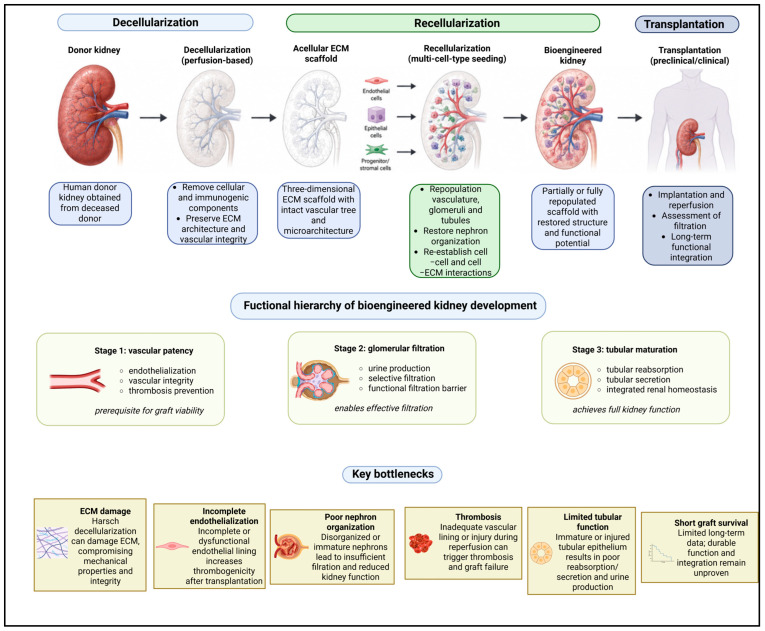
Workflow and functional hierarchy of kidney bioengineering using decellularization and recellularization approaches. Perfusion decellularization removes cellular and immunogenic elements from a human donor kidney while preserving the extracellular matrix (ECM), vascular architecture, and microstructure. This process yields a three-dimensional acellular ECM scaffold, which serves as a template for recellularization. The scaffold is repopulated with endothelial, epithelial, and progenitor/stromal cells to restore the vascular compartment, glomerular structures, and nephron architecture, and to re-establish cell–cell and cell–ECM interactions. The resulting bioengineered kidney can then be evaluated in transplantation models through implantation, reperfusion, filtration assessment, and long-term functional integration. The figure also illustrates a proposed hierarchical model of bioengineered kidney development consisting of three sequential functional stages: Stage 1, vascular patency, characterized by endothelialization, vascular integrity, and thrombosis prevention; Stage 2, glomerular filtration, characterized by urine production, selective filtration, and establishment of a functional filtration barrier; and Stage 3, tubular maturation, characterized by tubular reabsorption and secretion and the development of integrated renal function. Progression through these stages is sequential, with successful completion of earlier stages providing the foundation for subsequent functional maturation. Major translational challenges include: (i) ECM damage during decellularization; (ii) incomplete endothelialization resulting in thrombogenicity; (iii) poor nephron organization; (iv) thrombosis during reperfusion; (v) limited tubular function; and (vi) short graft survival with uncertain long-term functional durability.

The development of bioengineered kidneys can be conceptualized as the integration of three interdependent domains. These include decellularization, recellularization, and transplantation. Importantly, these domains do not develop simultaneously but sequentially. Each stage introduces distinct and increasingly complex challenges. The overall workflow of kidney bioengineering, including the major translational bottlenecks at each stage, is illustrated in Figure 1.

To recap, decellularization has been extensively studied, and the ECM structure can be preserved quite well through several protocols. Despite these advances, recellularization remains a major bottleneck. In particular, achieving complete epithelial coverage and nephron-level organization is challenging. In addition, transplantation introduces systemic issues, including thrombosis, immune responses, and long-term functional integration. However, despite extensive advances, there are still many discrepancies in the existing literature. For instance, there is a discrepancy regarding the relative influence of native ECM components versus matricellular growth factors in determining cell differentiation. Although numerous studies support the statement that the native ECM plays an instructive role by providing cues that direct stem cell fate, recent research has demonstrated that, when supplemented with the corresponding growth factors, synthetic scaffolds can achieve an equivalent level of differentiation. There may be a general preference for biochemical signals over native ECM structure.

Another dispute in the same vein concerns endothelial stability after transplantation. Re-endothelialization with HUVECs does improve graft vascular patency in the early stages. However, multiple studies indicate that these cells are rapidly lost and replaced by host-derived endothelium. This observation raises two crucial questions: is the function of seeded endothelial cells only a temporary anti-thrombotic barrier, or does stable engraftment of donor-derived endothelium ensure long-term graft function?

Lastly, the fundamental question of which cell source is best for recellularization remains unresolved. Multiple types of stem and progenitor cells have been used for recellularization. However, within the hierarchical model, different cell types contribute unequally to distinct functional stages: endothelial cells are essential for stage 1 (vascular patency), podocyte-like cells for stage 2 (glomerular filtration), and tubular epithelial cells for stage 3 (reabsorption and metabolic function). An important advantage of hiPSCs is their unlimited cell supply, which can be autologous in some cases, but concerns remain about tumorigenicity and incomplete differentiation. However, primary renal cells are more functionally specialized, but their availability and scalability are major issues. This trade-off between safety, functionality, and scalability poses a key challenge for clinical translation.

This framework suggests that future research should integrate strategies developed to simultaneously address vascularization, epithelial differentiation, and functional coupling. For example, advanced bioreactor systems combined with spatially controlled cell seeding and growth factor gradients may improve tissue organization. Such approaches could generate constructs that more closely resemble physiological kidney architecture prior to transplantation. Ultimately, the transition from proof-of-concept studies to clinically viable bioengineered kidneys depends on integrating these domains into a single reproducible and scalable platform. Further refinement of individual components alone will be insufficient. When all these aspects are thoroughly investigated, researchers should aim to reach a consensus on the optimal protocols for decellularization and recellularization. This would enable more reliable comparisons across studies and support valid conclusions, thereby advancing this technique toward clinical application.

Research involving hESCs remains ethically contentious because it requires the destruction of embryos, prompting persistent moral objections. By contrast, hiPSCs, reprogrammed from adult somatic cells, are generally considered ethically preferable because they avoid the destruction of embryos while retaining pluripotency, providing a potentially unlimited autologous cell source [19]. While these advantages are promising, there are important drawbacks to using hiPSCs as a cell source for scaffold repopulation. Tumorigenicity is a major concern with these cells compared with hESCs or renal progenitor cells. During somatic cell reprogramming, certain oncogenes can be activated, leading to genomic instability. In addition, iPSC-derived cell populations may include undifferentiated or partially reprogrammed cells that can form teratomas [82]. Efforts should be made to optimize this process.

As in every domain of tissue engineering for organ replacement, defining conclusive endpoints to ensure safety for human transplantation will be a major challenge [83]. Once the clinical application of bioengineered kidneys is feasible, other moral challenges will arise. In the early stages, treatment with bioengineered organs will be expensive, meaning that only those who can afford it will initially benefit. Consequently, this may further widen existing socioeconomic health disparities [84,85]. Studies on the socioeconomic impact of bioengineered kidneys are scarce. Development will entail substantial early-phase costs. Nevertheless, the ultimate goal is to make the process cost-effective over time compared with the lifelong costs of dialysis. As research advances toward the development of a complete human-sized bioengineered kidney, future research should aim to analyze the total cost of this process relative to current renal replacement therapies.

Although cell-on-scaffold kidneys may reduce or eliminate the need for immunosuppressive therapy by using autologous stem cells, they still rely on biological scaffolds derived from donor organs. Consequently, this approach does not inherently expand the donor pool. To address the limited availability of donor organs, 3D bioprinting has emerged as a promising strategy in tissue engineering. As demonstrated by Batchelder et al. [48], the decellularized biological scaffold can be replaced with a synthetic 3D-printed alternative, provided that appropriate growth and signaling factors are incorporated. This approach would provide an unlimited supply of scaffolds for subsequent cellular repopulation. 3D bioprinting of a fully functional kidney remains beyond current technical capabilities. Nevertheless, early successes have been reported, including the bioprinting of kidney organoids and tissue patches. These constructs have demonstrated partial functionality, including limited filtration and reabsorption [86]. A study [87] used a kidney-derived decellularized ECM as a bioink to fabricate 3D-printed renal constructs that demonstrated in vivo viability upon implantation in rat kidneys. Human kidney cells encapsulated in these constructs showed progressive maturation, as confirmed by expression of human-specific markers. The implanted constructs even displayed newly formed tubular- and glomerular-like structures.

Despite impressive advances in decellularization, recellularization, and transplantation of bioengineered kidneys, scientific feasibility alone will not suffice for clinical translation. Successfully bringing such technologies to the bedside will require overcoming a complex array of regulatory, manufacturing, safety, economic, and societal barriers. These issues take on added urgency because bioengineered kidneys, beyond being a combination of regenerative medicine and tissue engineering, are advanced therapy medicinal products (ATMPs) that encompass transplantation and cell-based therapeutics, placing them among the most highly regulated areas of contemporary medicine [35,36].

The first problem is regulatory classification. Bioengineered kidneys do not cleanly fit into the existing classification systems established for standard organ transplantation, medical devices, biologics, or cell therapies. In Europe, these products are most likely to be regulated as Advanced Therapy Medicinal Products within the European Medicine Agency’s regulatory framework, whereas in the USA, the regulatory authority is the Food and Drug Administration’s Center for Biologics Evaluation and Research (CBER) [35,37]. Determining the regulatory categorization of a bioengineered kidney is made very difficult when the end product comprises a decellularized donor scaffold, autologous or allogeneic stem-cell derivatives, biomaterials, growth factors, and ex vivo bioreactor conditioning. In short, future clinical application will depend on close cooperation among scientists, manufacturers, regulators, and transplant authorities to establish product-specific regulatory pathways and standardized release criteria.

Another key obstacle is producing products compliant with Good Manufacturing Practice (GMP). In fact, the protocols for bioengineered kidneys are currently very complicated, highly customized, and largely limited to research. Here, a clinical introduction would require the development of reproducible manufacturing pipelines capable of generating organs in strictly controlled, sterile environments, while maintaining genetic stability, cellular identity, and functional consistency [38,39]. This problem concerns not only the ability to expand and differentiate cells but also donor-organ procurement, scaffold decellularization, quality-control testing, bioreactor culture, transport logistics, and final product release. As a result, there is a need to create standardized manufacturing platforms that facilitate scalability and obtain regulatory approval.

Cell-source traceability is yet another major factor. It is necessary that each and every cellular element used in a bioengineered kidney can be fully traced from donor acquisition through transplantation. This means that, among other things, there must be records of donor eligibility, informed consent procedures, genetic characterization, culture history, differentiation protocols, cryopreservation conditions, and batch-specific quality controls (63). Traceability issues are even more relevant in induced pluripotent stem cell methods, where extended culture and extensive manipulation can lead to genetic or epigenetic changes. Genomic monitoring, including karyotyping, copy-number variation analysis, and whole-genome sequencing, might, as a result, evolve into standard components of future manufacturing pipelines [40,41].

The immunogenicity risks of bioengineered kidneys remain largely unknown. It is true that autologous hiPSC-derived cells are generally considered immunologically perfect; however, substantial evidence indicates that derivatives of induced pluripotent stem cells can still stimulate immune responses due to abnormal gene expression, epigenetic memory, mitochondrial changes, neoantigens, or incomplete differentiation [26,27]. In addition, residual donor-derived extracellular matrix proteins within the decellularized scaffolds may retain antigenic properties that can elicit immune responses post-transplantation. Therefore, detailed immunologic profiling will be essential before such therapies can be used clinically, even when using autologous cell sources.

Besides immunogenicity, tumorigenicity remains one of the biggest safety concerns. Pluripotent stem cells have a limitless capacity to self-renew and can develop into teratomas if undifferentiated cells remain in the graft [42,88]. In addition, long-term in vitro culture can lead to the accumulation of chromosomal changes, cancer-related mutations, and epigenetic changes that may increase cell proliferation. Therefore, regulatory bodies may require both in vitro and in vivo studies demonstrating the absence of residual pluripotent cells, genomic instability, malignant transformation, and uncontrolled proliferation before granting permission for human trials [36,42]. Two essential components of the translational pipeline will likely be standardized methods for tumorigenicity testing and long-term animal models.

Long-term follow-up requirements add to the challenge. Bioengineered organs, unlike regular pharmaceuticals, are designed to function for decades, so safety evaluation should not be limited to short-term findings. Long-term follow-up will be essential to assess how well the organ holds up, identify chronic inflammation, fibrosis, vascular changes, immune responses, possible cancerous changes, and ongoing physiological performance [35,36]. Cell and gene therapy regulations already recommend extending post-marketing surveillance for more than 10–15 years in some cases, and bioengineered organs may fall under similar guidelines [37].

Equity issues in access must be considered from the outset of product development. Personalized bioengineered kidneys will be expensive at first because of the need for specialized labs, advanced manufacturing facilities, and substantial investment. If affordability and accessibility are not prioritized, these technologies could exacerbate disparities in access to transplantation and renal replacement therapy [89]. Economic analysis must play a central role in technology development. It must move beyond manufacturing costs to also examine reimbursement policies, the sustainability of the health system, global accessibility, and fair distribution methods. At the end of the day, the evaluation of bioartificial kidneys ought to go beyond the scientific discoveries they have brought about and also assess their ability to provide safe, reliable, and widely accessible treatment for patients with kidney failure.

These points collectively show that the road to the clinic consists of much more than simply overcoming biological and engineering problems. Progress will next depend on the parallel development of regulatory benchmarks, GMP-compliant manufacturing plants, safety evaluation approaches, continuous monitoring schemes, and equitable access models that will allow the responsible introduction of bioengineered kidneys in clinical settings.

Certain limitations of this narrative review should be acknowledged. The literature search and article selection were based on thematic relevance rather than strict inclusion criteria, which may introduce selection and publication bias. The synthesis of current findings relies on interpreting heterogeneous preclinical studies that differ in experimental models, cell types, and outcome measures. Consequently, the conclusions should be viewed as an integrative overview rather than definitive evidence.

## 5. Conclusions

Tremendous advancements have been made in kidney bioengineering. The most important finding is that the kidney develops in a hierarchical model, with vascularization, glomerular filtration, and tubule maturation occurring in sequence and reflecting increasing complexity. Among these steps, vascular stability is the key factor determining early success, while the primary barrier to complete functional recovery is insufficient epithelial cell maturation. For this reason, future research ought to be directed first and foremost toward programs that build up the endothelial lining and induce epithelial cell differentiation, rather than merely increasing cellular diversity. In the absence of addressing these stage-specific bottlenecks, increasing technical complexity is unlikely to yield clinically relevant improvements.

## Figures and Tables

**Table 1:** Decellularization and recellularization strategies in kidney bioengineering and their functional outcomes.

Study	Model	Implantation Site	Observation Period	Anticoagulation Strategy	Recellularization	Urine Production	Creatinine/Urea Clearance	Thrombosis Outcome	Survival Duration	Main Findings
Song et al. (2013) [28]	Rat	Orthotopic	Short-term (hours)	Heparin (reported)	HUVECs (renal artery) + neonatal kidney cells (ureter)	Yes	Present but markedly reduced compared with native kidney	Partial vascular patency; thrombosis remained a concern	Hours	First demonstration of orthotopic transplantation of a recellularized kidney; rudimentary urine production and partial filtration
Peloso et al. (2015) [50]	Rat	Orthotopic	7 days	NR	None (acellular scaffold)	No	No	Complete vascular thrombosis	7 days	Structural preservation and successful implantation, but no functional activity due to thrombosis
Orlando et al. (2012) [29]	Pig	Orthotopic	14 days	NR	None (acellular scaffold)	No	No	Universal thrombosis of renal vessels	14 days	Preserved scaffold architecture and successful reperfusion, but complete vascular occlusion
Kajbafzadeh et al. (2019) [51]	Sheep	Orthotopic	2–12 h	NR	None (acellular scaffold)	No	No	Rapid thrombosis and vascular leakage	2–12 h	Demonstrated surgical feasibility and impact of decellularization protocol on vascular integrity
Uzarski et al. (2023) [30]	Pig	Orthotopic	Up to 7 days	Heparin + antiplatelet therapy	HUVECs (arterial and venous perfusion)	No	No	Sustained vascular patency; thrombosis in subset of grafts	Up to 7 days	First large-animal demonstration of prolonged vascular patency after endothelialization
Lo et al. (2024) [31]	Pig	Orthotopic	Short-term (hours)	Heparin + antiplatelet therapy	HUVECs (vascular compartment) + glomerular outgrowth cells (ureter)	Yes	Evidence of filtration; quantitative clearance remained limited	Reduced thrombosis compared with acellular controls	Hours	Urine production and selective filtration demonstrated in a large-animal model
Geng et al. (2022) [52]	Rat	Orthotopic	Immediate/short-term	NR	iPSC-derived nephron progenitor cells + ECM modification (Nap-FF-GRGD)	NR	NR	NR	Short-term	Improved cell adhesion and nephron-like organization after transplantation

Abbreviations: HUVECs, human umbilical vein endothelial cells; iPSC, induced pluripotent stem cell; ECM, extracellular matrix.

**Table 2:** Cell types for recellularization of bioengineered kidney scaffolds: functional roles, advantages, limitations, and translational relevance.

Cell Type	Primary Target Structure	Functional Role in Bioengineered Kidney	Key Advantages	Key Limitations and Challenges	Translational Status	Refs.
HUVECs (Human umbilical vein endothelial cells)	Vascular tree (arterial, venous, glomerular capillaries)	Rapid endothelialization to restore vascular patency and reduce thrombogenicity	Easily obtainable; no differentiation required; well-characterized endothelial model	Limited proliferative lifespan; poor long-term engraftment; partial detachment and replacement by host endothelium in vivo	Widely used in large-animal transplantation models	[30,31,32]
hiPSCs (Human induced pluripotent stem cells)	Multiple renal compartments (vascular, tubular, glomerular)	Differentiation into endothelial, epithelial, and stromal lineages	Autologous potential; unlimited expansion capacity; avoids ethical concerns of embryonic sources	Risk of tumorigenicity (teratoma formation); incomplete or heterogeneous differentiation; genomic instability; need for stringent purification	Preclinical (in vitro and small/large animal models)	[32,48,82]
hESCs (Human embryonic stem cells)	Multiple renal compartments	Pluripotent differentiation into renal progenitor and mature cell types	High differentiation efficiency; robust pluripotency; well-characterized developmental pathways	Ethical concerns; risk of teratoma formation; immunogenicity (non-autologous)	Preclinical (in vitro and animal models)	[48,81]
Nephron progenitor cells (NPCs)	Developing nephron structures (glomeruli and tubules)	Differentiation into nephron components, including podocytes and tubular cells	Kidney-specific lineage commitment; enhanced capacity for nephron reconstruction	Limited availability; difficult expansion; incomplete maturation after transplantation	Preclinical (primarily rodent models)	[52,81]
Neonatal kidney cells (NKCs)	Mixed nephron compartments	Partial reconstruction of nephron-like structures; support early functional integration	Contain heterogeneous renal cell populations; relatively high plasticity	Limited to animal-derived sources; variability; poor scalability; ethical and translational constraints	Preclinical (rodent transplantation models)	[28]
Nephrosphere-derived cells	Tubular and glomerular compartments	Differentiation into epithelial and endothelial renal cell types depending on microenvironment	Capable of multilineage differentiation; responsive to culture conditions	Limited yield from donor tissue; phenotypic instability; incomplete functional maturation	Preclinical (human-tissue-based in vitro models)	[49]
GOCs (Glomerular outgrowth cells)	Glomeruli (podocyte layer)	Podocyte differentiation and contribution to size-selective glomerular filtration barrier	Demonstrated contribution to filtration function in large-animal models; relevant for glomerular physiology	Complex isolation and expansion; incomplete maturation; limited availability; specialized differentiation protocols required	Large-animal transplantation models	[31]
hiPSC-derived endothelial cells (hiPSC-ECs)	Microvascular and macrovascular endothelium	Re-endothelialization with improved adhesion and coverage compared to HUVECs	Potential for autologous use; improved endothelial stability; scalable	Differentiation complexity; variability in phenotype; requires optimization of culture conditions	Preclinical (human scaffold studies)	[32]

Abbreviations: HUVECs, human umbilical vein endothelial cells; hiPSCs, human induced pluripotent stem cells; hESCs, human embryonic stem cells; NPCs, nephron progenitor cells; NKCs, neonatal kidney cells; GOCs, glomerular outgrowth cells; hiPSC-ECs, human induced pluripotent stem cell-derived endothelial cells.

## Data Availability

No new data were created or analyzed in this study.

## Abbreviations

The following abbreviations are used in this manuscript:

CKD	Chronic kidney disease
ECM	Extracellular matrix
RRT	Renal replacement therapy
CTA	CT-angiography
SDS	Sodium dodecyl sulfate
PBS	Phosphate-buffered solution
SLES	Sodium lauryl ether sulfate
hUC-MSC	Human umbilical cord mesenchymal stromal/stem cells
hiPSC	Human induced pluripotent stem cell
hESC	Human endothelial stem cell
HUVEC	Human umbilical vein endothelial cell
NKC	Neonatal kidney cell
GOC	Glomerular outgrowth cell
Nap-FFGRGD	Naphthalenephenylalanine-phenylalanine-glycine-arginine-glycine-aspartic
hiPSC-EC	Human induced pluripotent stem cell-derived endothelial cell
VEGF	Vascular endothelial growth factor
CBP	Collagen binding peptide
